# The Effect of an App for Day-to-Day Postoperative Care Education on Patients With Total Knee Replacement: Randomized Controlled Trial

**DOI:** 10.2196/15323

**Published:** 2019-10-21

**Authors:** Thomas Timmers, Loes Janssen, Walter van der Weegen, Dirk Das, Willem-Jan Marijnissen, Gerjon Hannink, Babette C van der Zwaard, Adriaan Plat, Bregje Thomassen, Jan-Willem Swen, Rudolf B Kool, Frederik Okke Lambers Heerspink

**Affiliations:** 1 Interactive Studios Rosmalen Netherlands; 2 Radboud university medical center, Radboud Institute for Health Sciences, IQ healthcare Nijmegen Netherlands; 3 Máxima MC Veldhoven Netherlands; 4 Sint Anna Hospital Geldrop Netherlands; 5 Albert Schweitzer Hospital Dordrecht Netherlands; 6 Radboud university medical center Nijmegen Netherlands; 7 Jeroen Bosch Hospital 's-Hertogenbosch Netherlands; 8 Haaglanden Medical Center 's-Gravenhage Netherlands; 9 VieCuri Medical Centre Venlo Netherlands

**Keywords:** patient education, postoperative care, smartphone, self-management, ehealth, telemedicine

## Abstract

**Background:**

Patients who undergo primary Total Knee Replacement surgery (TKR) are often discharged within 1-3 days after surgery. With this relatively short length of hospital stay, a patient’s self-management is a crucial factor in optimizing the outcome of their treatment. In the case of TKR, self-management primarily involves adequate pain management, followed by physiotherapy exercises and daily self-care activities. Patients are educated on all these topics by hospital staff upon discharge from the hospital but often struggle to comprehend this information due to its quantity, complexity, and the passive mode of communication used to convey it.

**Objective:**

This study primarily aims to determine whether actively educating TKR patients with timely, day-to-day postoperative care information through an app could lead to a decrease in their level of pain compared to those who only receive standard information about their recovery through the app. In addition, physical functioning, quality of life, ability to perform physiotherapy exercises and daily self-care activities, satisfaction with information, perceived involvement by the hospital, and health care consumption were also assessed.

**Methods:**

A multicenter randomized controlled trial was performed in five Dutch hospitals. In total, 213 patients who had undergone elective, primary, unilateral TKR participated. All patients had access to an app for their smartphone and tablet to guide them after discharge. The intervention group could unlock day-to-day information by entering a personal code. The control group only received weekly, basic information. Primary (level of pain) and secondary outcomes (physical functioning, quality of life, ability to perform physiotherapy exercises and activities of daily self-care, satisfaction with information, perceived involvement by the hospital, and health care consumption) were measured using self-reported online questionnaires. All outcomes were measured weekly in the four weeks after discharge, except for physical functioning and quality of life, which were measured at baseline and at four weeks after discharge. Data was analyzed using Student *t* tests, chi-square tests, and linear mixed models for repeated measures.

**Results:**

In total, 114 patients were enrolled in the intervention group (IG) and 99 in the control group (CG). Four weeks after discharge, patients in the IG performed significantly better than patients in the CG on all dimensions of pain: pain at rest (mean 3.45 vs mean 4.59; *P*=.001), pain during activity (mean 3.99 vs mean 5.08; *P*&lt;.001) and pain at night (mean 4.18 vs mean 5.21; *P*=.003). Additionally, significant differences were demonstrated in favor of the intervention group for all secondary outcomes.

**Conclusions:**

In the four weeks following TKR, the active and day-to-day education of patients via the app significantly decreased their level of pain and improved their physical functioning, quality of life, ability to perform physiotherapy exercises and activities of daily self-care, satisfaction with information, perceived involvement by the hospital, and health care consumption compared to standard patient education. Given the rising number of TKR patients and the increased emphasis on self-management, we suggest using an app with timely postoperative care education as a standard part of care.

**Trial Registration:**

Netherlands Trial Register NTR7182; https://www.trialregister.nl/trial/6992

## Introduction

### Background

Osteoarthritis of the knee is one of the leading causes of disability among adults aged 65 years old and over [1]. Complaints often result in a loss of productivity and reduced quality of life [2,3]. Total Knee Replacement (TKR) is considered the best available treatment option when conservative options have failed, resulting in a postoperative relief of pain, increased functional outcomes, and a patient satisfaction of more than 80% [4]. TKR is one of the most commonly performed orthopedic procedures internationally and in the United States alone its rate of occurrence is estimated to increase to more than 3.4 million procedures by the year 2030 [3].

In the last decade, TKR fast-track (or enhanced recovery) pathways have been implemented in many hospitals. These pathways imply a combination of patient education, multimodal analgesia, early mobilization, fluid management and nutrition optimization [5,6]. These fast-track pathways have led to a substantial decrease in patients’ length of hospital stay following TKR, which reduced, on average, from 10-11 days in 2000 to 2-4 days in 2013 [7]. Nowadays, TKR is sometimes even performed as a one-day procedure in which patients do not need to stay overnight in the hospital [8]. Time to full recovery after TKR takes an average of 6-12 months.

Because of the shortened length of stay, patients’ self-management has become a crucial factor in optimizing their health outcomes. According to the World Health Organization, postoperative self-management is the ability of individuals, families, and communities to cope with illness, with or without the support of a health care provider [9]. In the case of TKR, postoperative self-management primarily involves controlling the level of pain, followed by performing physiotherapy exercises and daily self-care activities [10-13]. Next to the available information in brochures, hospital staff educate patients on these topics to prepare them on how best to manage their new situation when back at home.

Patients often struggle to comprehend this information due to its quantity, complexity, and the passive mode of communication used to convey it [14,15]. This leads to a limited amount of knowledge and confidence regarding self-management [16,17], which is a predictor for lower adherence rates [18]. Additionally, patients’ pain-related fear of movement, also referred to as kinesiophobia, is known to negatively affect TKR early outcomes [19]. This leads to lower rates of satisfaction, as patients feel the discharge process is rushed and that they are no longer being cared for until the next follow-up, which is usually six to eight weeks after discharge [16].

Electronic health (eHealth) or mobile health (mHealth) offer a potentially powerful means for active patient education and behavioral change reinforcement [20]. A 2018 review on perioperative eHealth interventions has demonstrated a positive effect upon the postoperative course in patients who are undergoing orthopedic and cardiac surgery [21]. The interventions described in the review range from an educational website to the facilitation of telemonitoring and teleconsultation. However, evidence concerning the use of smartphone or tablet apps is scarce. This is surprising given the number of people that own a mobile device, coupled with the increase in availability and usage of medical mobile applications [22,23]. Additionally, smartphones and tablets possess the unique ability to receive push-notifications that can be used to actively inform patients at times when the information becomes relevant for them. These notifications can provide patients with personalized guidance at various stages of their patient journey.

### Objectives

The aim of this randomized controlled trial was to investigate the effect of an interactive app on patients’ level of pain, physical functioning, quality of life, satisfaction, and health care consumption in the first four weeks of recovery after TKR. We hypothesized that, compared to standard practices of patient education, providing patients with timely, day-to-day information via an app would have a positive effect on all outcomes.

The primary outcome of the study was patients’ level of pain during the first four weeks after discharge. The secondary outcomes of the study were physical functioning, quality of life, patients’ ability to perform physiotherapy exercises and daily self-care activities, satisfaction with the information provided, perceived level of postoperative involvement by the hospital, and health care consumption. All these outcomes were measured by means of self-reported, online questionnaires.

## Methods

### Study Design

A total of five Dutch hospitals (four nonacademic teaching hospitals and one general hospital) participated in the study. Between May and December 2018, patients scheduled for elective, primary, unilateral TKR were invited to participate in a surgeon-blinded, randomized controlled trial. The study focused on the four-week period following discharge from the hospital and assessed the effectiveness of an interactive app compared to the standard of care in a parallel group design with equal allocation ratio. No changes were made to the design after the study was commenced. We followed the Consolidated Standards of Reporting Trials (CONSORT) guidelines and the CONSORT EHEALTH Checklist [24,25].

### Informed Consent and Ethical Considerations

Consent was gained via hospital staff who contacted patients by phone to ask them to consider participation in the study about two weeks prior to their TKR. Patients willing to participate received an email with all the necessary study information required for informed consent. Patients were offered at least two days to reflect on the information. In case of any questions, patients could contact the local research coordinator by phone or by email. Patients gave consent by signing an online informed consent form. There were no indicators of substantial risk as a function of participating in this study. The study was registered at the Netherlands Trial Registry (NTR), with the reference number 6992. The study was approved by the Institutional Review Board of the Maxima Medical Centre (Eindhoven, The Netherlands) with the reference number N17.158.

### Participant Selection

Patients scheduled for elective, primary, unilateral TKR and aged 40 years and above were eligible for inclusion. Additionally, participants were required to be fluent in Dutch and in the possession of an email address and smartphone or tablet.

### Intervention

The Patient Journey App (Interactive Studios, Rosmalen, The Netherlands) was used as the intervention to provide information to both patient groups. All patients had access to the app to guide them after discharge. The control group only received basic information about the recovery process about two times per week. The intervention group could unlock day-to-day information by entering a personal code. Participants in the intervention group received this personal code by email after completing the baseline questionnaire.

All patients in the intervention group received the same information via the app. However, aligning the timing of the information to the individual patient’s phase of recovery gave it a personalized character. Patient’s date of discharge was used to assure the timing of the information was correct. Push notifications were used to actively alert patients about information being available. The timing of the push notifications was configured per information item (eg, information about pain medication was available 1 day after discharge at 11 am and information about physiotherapy exercises was available 2 days after discharge at 2 pm).

**Figure 1:**
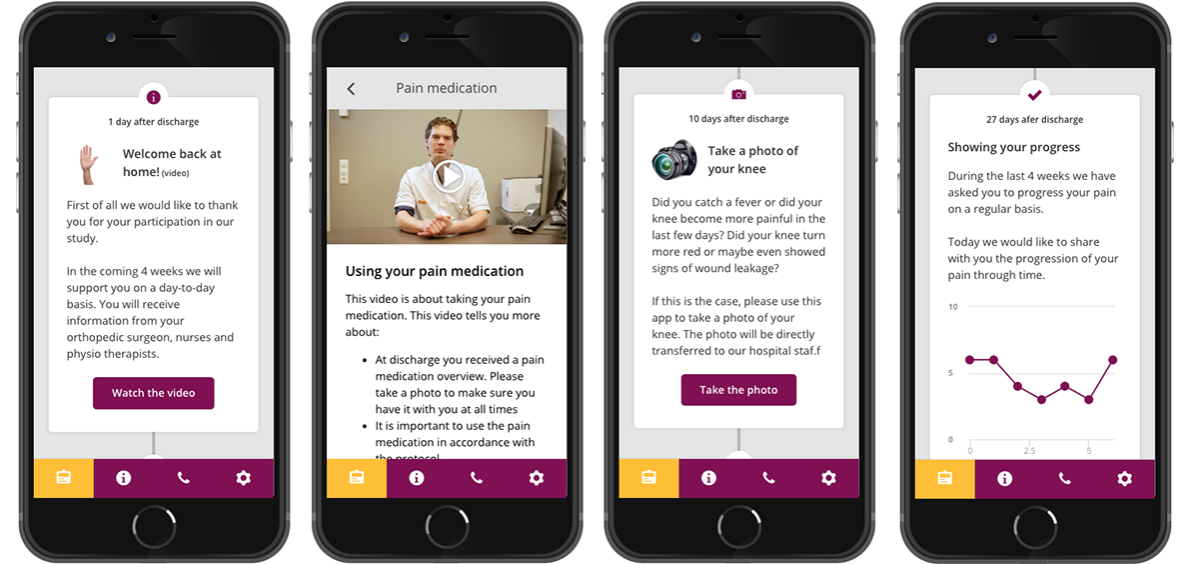
Examples of the interactive app used as an intervention, translated from Dutch (language used in the study) to English. From left to right: the welcoming of patients to the app, video and text information about medication usage, an invitation to send a photo of the wound (in case of fever, increased levels of pain or wound leakage) and a patient-reported pain score progress tracker.

The text, photos, and videos that patients in the intervention group received were developed specifically for this trial and composed and based upon interviews with orthopedic surgeons, physician assistants, nurses, and physiotherapists from participating hospitals. Furthermore, electronic health records of 50 patients who had previously undergone TKR were checked to determine for what reasons they had contacted the hospital. Based on this information, an interactive timeline was developed (Figure 1). This information was not available to patients in the control group. All information on the timeline was presented in Dutch.

Information in the app was tailored for each hospital and based on existing protocols. During the 28-day period after discharge, every patient in the intervention group received over 30 notifications with supporting information, related to topics such as pain, physiotherapy exercises, wound care, and daily self-care activities (see Multimedia Appendix 1). Apart from the information being available in the app at the timeline, it was also available as an alphabetically ordered reference within the app. Additionally, patients were requested to enter their pain scores on a weekly basis and were able to check their results within an interactive graph every week. Patients also had the opportunity to upload a photo of the wound in case of fever and an increase in pain or wound leakage. Prior to study initiation, six TKR patients at two of the participating hospitals were interviewed to generally assess the usefulness and usability of the app. They reported that the app would be very useful and had no suggestions for changes. After the study, all the content developed for the intervention was provided to the participating hospitals, allowing them to partially offer it to their patients as the new standard of care.

### Study Outcomes

As the primary outcome, the patients’ ability to manage their pain was assessed in the four weeks following discharge from the hospital. As secondary outcomes, we assessed patients’ physical functioning and quality of life at baseline and four weeks after discharge. Additionally, patients’ ability to perform physiotherapy exercises and daily self-care activities, satisfaction with the information provided, perceived level of postoperative involvement by the hospital, and health care consumption were assessed weekly during the four weeks following discharge (Table 1). Finally, data on app usage was continuously captured to get a better understanding of how the app was being used over time, the type of information that patients consulted, and the videos they watched.

**Table 1:** Overview of used questionnaires per outcome.

Outcome	Questionnaire
Level of pain^a^	Three questions concerning pain while at rest, during activity and during the night. NRS^b^ scores were used to measure the outcome, ranging from 0 (no pain at all) to 10 (worst pain imaginable).
Physical functioning^a^	The Knee injury and Osteoarthritis Outcome Score short form [26] involves 7 multiple choice questions indicating functional limitations, ranging from 0 (minimal limitations) to 100 (maximal limitations).
Quality of life^a^	EuroQol 3-level questionnaire, EQ-5D-3. Measuring 5 dimensions (mobility, self-care, usual activities, pain/discomfort, anxiety depression) on a 3-level scale (no problems, some problems, extreme problems). Additionally, a VAS^c^ was used to assess patients self-rated health. VAS endpoints range from 0 (Worst imaginable state) to 100 (Best imaginable state) [27].
Physiotherapy exercises (developed for this study)	One question concerning patients’ ability to perform their physiotherapy exercises. NRS score was used to measure the outcome, ranging from 0 (not capable at all) to 10 (perfectly capable).
Daily self-care activities (developed for this study)	One question concerning patients’ ability to perform their daily self-care activities. NRS score was used to measure the outcome, ranging from 0 (not capable at all) to 10 (perfectly capable).
Satisfaction with information (developed for this study)	One question concerning patients’ satisfaction about the information they received from the hospital after discharge. NRS score was used to measure the outcome, ranging from 0 (not satisfied at all) to 10 (very much satisfied).
Perceived involvement (developed for this study)	One question concerning the patient-perceived level of involvement by the hospital postoperatively. NRS score was used to measure the outcome, ranging from 0 (not satisfied at all) to 10 (very much satisfied).
Health care consumption (developed for this study)	Three dichotomized questions concerning health care consumption at the hospital, the general practitioner and home-care organization. When a patient indicated they had contacted one of these organizations, the outcome was assessed (ie, no action required or follow-up action required). If follow-up was required, that could entail things like a consultation or referral to another organization.

^a^These questionnaires are part of the Patient Reported Outcome Measures guidelines of the Dutch Orthopedic Association (Nederlandse Orthopaedische Vereniging, NOV) for TKR.

^b^NRS: numeric rating scale.

^c^VAS: visual analogue scale.

Some questions were developed especially for this study. These questionnaires were checked by surgeons and researchers from participating hospitals (see Multimedia Appendix 2). Additionally, a specialist organization on using acceptable language (Bureau Beter Taal, Beusichem, The Netherlands) reviewed and revised the questionnaires to assure readability for about 95% of the Dutch population (language level B1). Finally, the questionnaires were tested for usability by six patients in two of the participating hospitals prior to study initiation, which lead to no additional changes.

Study outcomes were measured five times in total: at baseline and on a weekly basis in the four weeks after discharge (Table 2). The baseline measurement was taken directly after patients were first included in the study. Follow-up questionnaires were sent to both groups at the end of each postoperative week, allowing patients to reflect on each previous week. Per measurement, a maximum of two email reminders were sent in case a patient would not respond. To minimize the risk of recall bias, patients only had a four-day time period to complete the questionnaires for each measurement. All outcome data was self-reported and collected using an online system. Patients who either missed the baseline measurement or more than two of their follow-up questionnaires were registered as lost to follow-up. These patients were not included in the analysis.

**Table 2:** Overview of outcomes per time point measurement.

Baseline	Follow-up week 1, 2 and 3	Follow-up week 4
Patient characteristics	–^a^	–
Physical functioning	–	Physical functioning
Quality of life	–	Quality of life
–	Level of pain	Level of pain
–	Physiotherapy exercises	Physiotherapy exercises
–	Daily self-care activities	Daily self-care activities
–	Satisfaction with information	Satisfaction with information
–	Perceived involvement	Perceived involvement
–	Health care consumption	Health care consumption

^a^Not applicable.

### Sample Size

The sample size calculation was based on the 2018 Hardt et al study [28] in which an app was used postoperatively to educate patients on physiotherapy exercises and pain management. In this study a 1.0-point difference in the level of pain (on a numeric rating scale [NRS] of 0-10) was found in favor of the intervention group. Since this intervention was used in a hospital setting with nurses controlling for the right application of the intervention, we expect the effect or our intervention to be lower.

We performed an *a priori* sample size calculation (alpha=.05, [1−beta] =.90), based on a repeated measures analysis of variance (ANOVA) (groups=2, measurements=4) and an effect size of 0.25 that was based on a between-groups difference of 0.5 (SD 1.0). This calculation resulted in a minimum requirement of 78 patients in each study arm. Adding an expected loss to follow-up of 20% led to a total sample size of 190 patients. The sample size calculation was performed using G*Power, version 3.1 (Universität Düsseldorf, Düsseldorf, Germany).

### Randomization

Patients were randomized by a computer to either a control or intervention group. Randomization was performed without block or stratification restrictions. After being allocated to one of the groups, patients received an email that included the link to the online informed consent form and the baseline questionnaire.

### Statistical Methods

For our primary analysis we used an intention-to-treat approach including all randomized patients. Normally distributed continuous variables (eg, physical functioning and quality of life) were presented as a mean value with an SD and were statistically compared between the groups using independent Student two-tailed *t* tests. Nonnormally distributed variables were presented as a median value with the interquartile range. Categorical variables (eg, health care consumption) were presented as number and percentage and compared between groups using Chi-square tests. Linear mixed models for repeated measures were used to estimate the effect of the use of the intervention, using primary and secondary outcomes (ie, level of pain, performing physiotherapy exercises, performing activities of daily self-care, satisfaction with information, and patient-perceived involvement by the hospital) as dependent variables and intervention group, time, and the interaction between time and intervention group as fixed effect variables. Patient ID and location were used as random effect variables. Missing data were not replaced in any type of analysis.

Patients’ level of education was split into two groups for the purpose of analysis: group 1 (none, elementary school, and secondary [vocational] education) and group 2 (higher secondary education, preuniversity education, and university [of applied science]). *P*≤.05 was assumed to indicate a significant difference. *P* values between .05 and .10 were assumed to indicate a trend. As per protocol, analysis was performed to examine the robustness of our results by specifically analyzing the results of patients in the intervention group that downloaded and used the app. All data was analyzed using SPSS version 25.0, (IBM, Armonk, United States), except for the linear mixed model analysis which was executed using R, version 3.6.0 (R Foundation for Statistical Computing, Vienna, Austria).

## Results

### Study Sample

**Figure 2:**
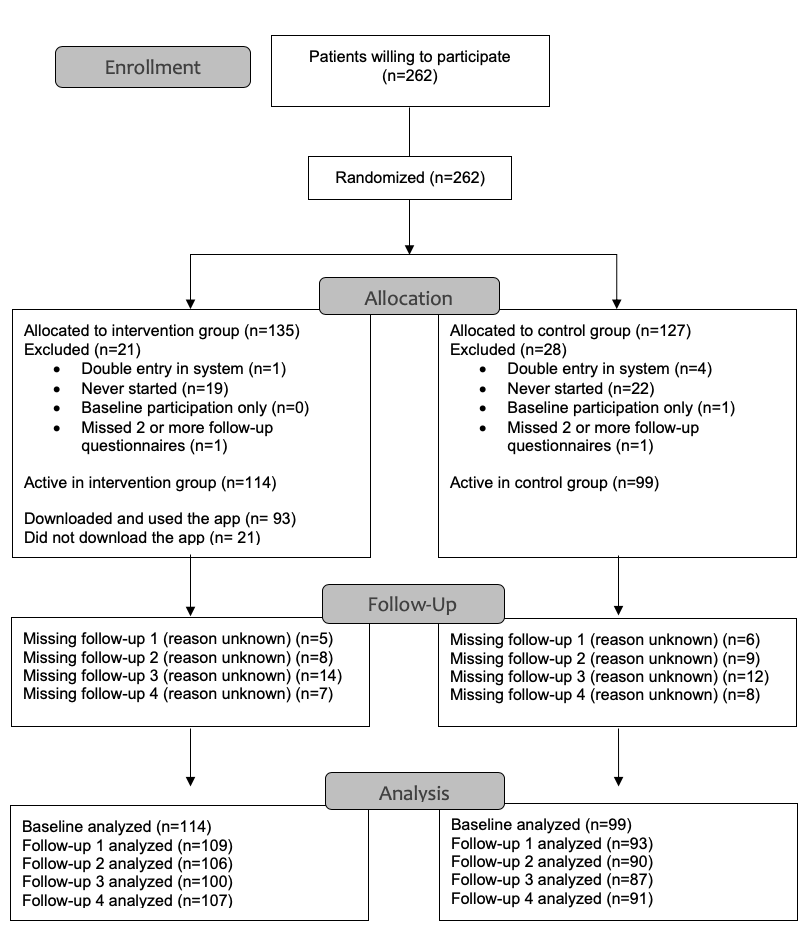
Patient flow diagram.

Between May and December 2018, a total of 262 eligible patients were willing to participate in the study. A total of 41 patients (15.6%) withdrew from the study without completing the baseline questionnaire (for reasons unknown). One patient (0.4%) only completed the baseline questionnaire, and an additional two patients (0.8%) did not complete more than two follow-up questionnaires. In total, 114 patients were actively enrolled in the intervention group and 99 patients in the control group. In the intervention group, 93 patients downloaded and used the app (Figure 2).

Baseline characteristics of the study population were largely similar between groups (Table 3).

**Table 3:** Patient characteristics.

Characteristics		Intervention group (n=114)	Control group (n=99)
**Sex, n (%)**			
	Male	40 (35.1)	39 (39.4)
	Female	74 (64.9)	60 (60.6)
Age (years), mean (SD)		64.74 (7.57)	65.63 (7.90)
**Education, n (%)**			
	Group 1	52 (46.0)	47 (47.5)
	Group 2	61 (54.0)	52 (52.5)
**Home-situation, n (%)**			
	Living alone	27 (23.7)	21 (21.2)
	Living together	87 (76.3)	78 (78.8)
KOOS PS^a^, mean (SD)		45.27 (12.71)	44.05 (11.10)
**Quality of Life**			
	EQ-5D-3, mean (SD)	0.67 (0.21)	0.65 (0.24)
	EQ-5D-3 VAS^b^, mean (SD)	69.59 (16.38)	67.42 (19.56)

^a^KOOS PS: Knee injury and Osteoarthritis Outcome Score short form.

^b^VAS: visual analogue scale.

### Primary Outcomes

#### Level of pain

**Figure 3:**
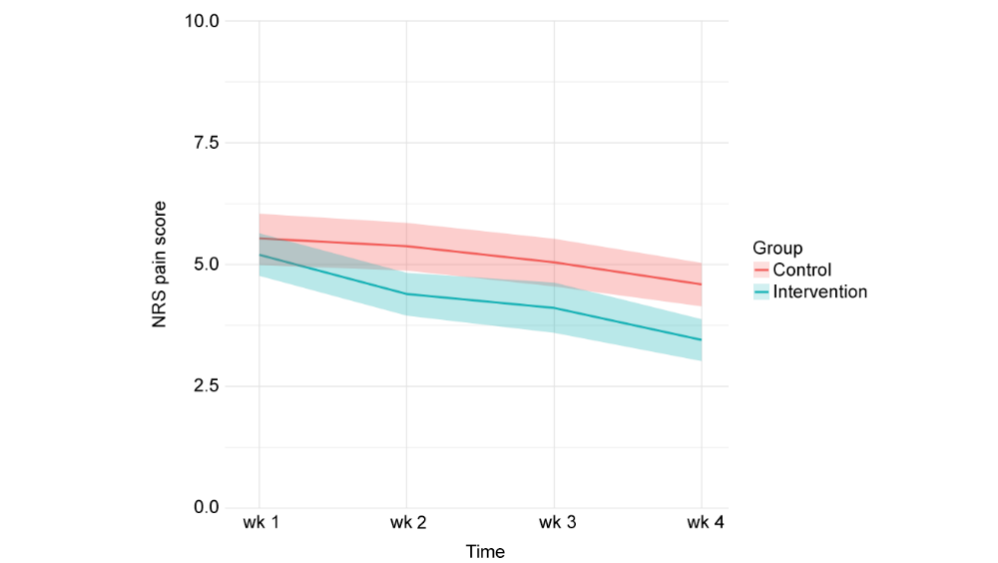
Pain at rest (measured weekly in the first four weeks after discharge, 95% CI).

**Figure 4:**
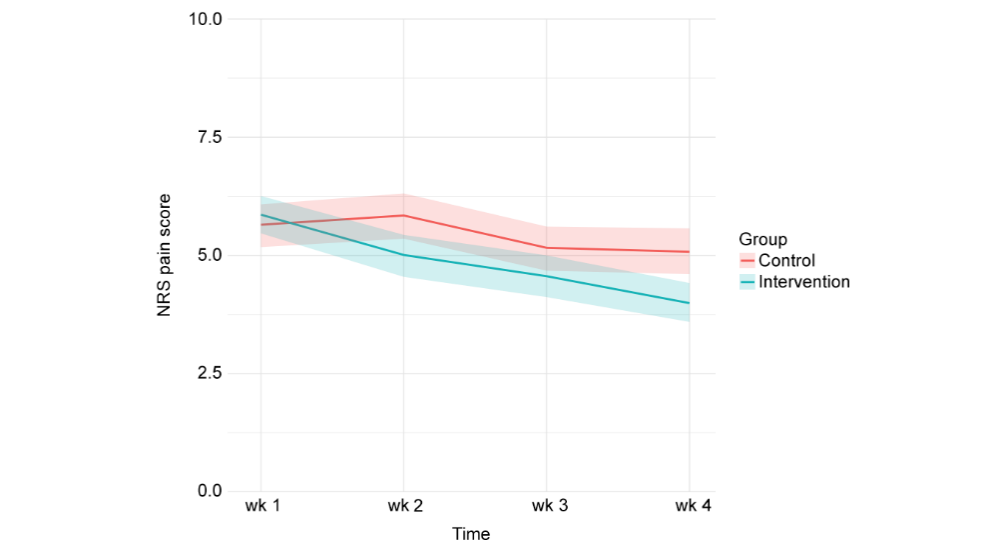
Pain during activity (measured weekly in the first four weeks after discharge, 95% CI).

**Figure 5:**
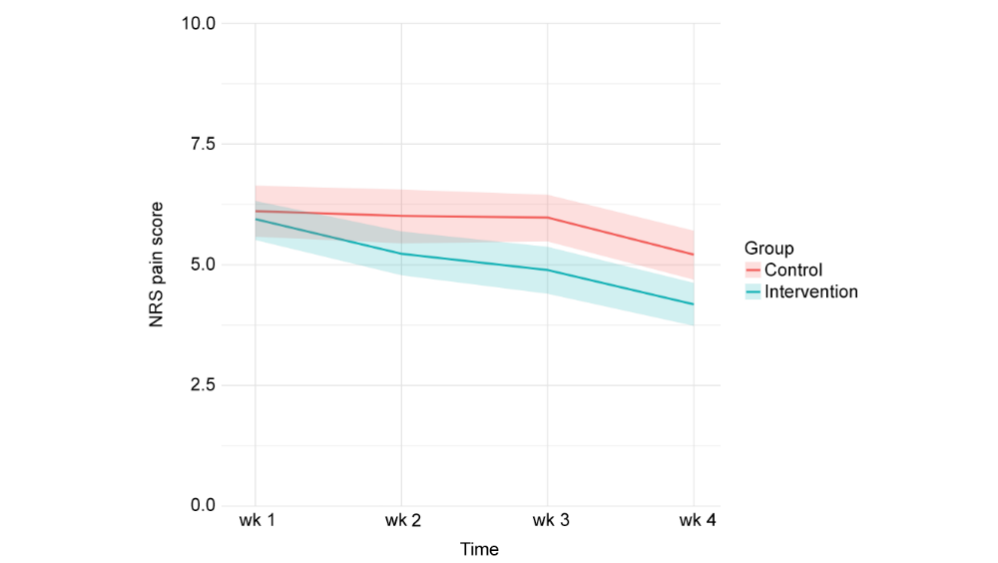
Pain at night (measured weekly in the first four weeks after discharge, 95% CI).

In both the intervention and control group, patients’ levels of pain decreased on all three dimensions of pain during the first four weeks after discharge (Figure 3, Figure 4, and Figure 5). Patients in the intervention group performed better on each of the dimensions from the second week onwards (Table 4).

**Table 4:** Level of pain.

Wk	Pain at rest				Pain during activity				Pain during the night			
	Intervention group, mean	Control group, mean	Model estimated difference (95% CI)	*P* value	Intervention group, mean	Control group, mean	Model estimated difference (95% CI)	*P* value	Intervention group, mean	Control group, mean	Model estimated difference (95% CI)	*P* value
1	5.20	5.54	0.25 (–0.41 to 0.90)	.46	5.86	5.65	–0.20 (–0.82 to 0.42)	.52	5.94	6.11	0.08 (–0.58 to 0.74)	.81
2	4.40	5.38	0.96 (0.31 to 1.62)	.004	5.01	5.84	0.81 (0.19 to 1.43)	.01	5.23	6.01	0.80 (0.14 to 1.46)	.02
3	4.11	5.05	0.88 (0.21 to 1.54)	.001	4.56	5.16	0.60 (0.03 to 1.23)	.06	4.89	5.98	1.05 (0.37 to 1.72)	.002
4	3.45	4.59	1.07 (0.42 to 1.73)	.001	3.99	5.08	1.11 (0.48 to 1.73)	<.001	4.18	5.21	1.02 (0.36 to 1.68)	.003

### Secondary Outcomes

#### Physical Functioning and Quality of Life

Four weeks after discharge, patients in the intervention group reported a significant, 14% decrease in functional limitations (Knee injury and Osteoarthritis Outcome Score short form [KOOS PS]) compared to the control group (*P*<.001) (Table 5). With respect to quality of life (EQ-5D), patients in the intervention group also reported a significant, 14% increase compared to the control group (*P*<.001).

**Table 5:** Physical functioning and quality of life.

		Baseline	Week 4
**Physical functioning**			
	Intervention group, mean (SD)	45.91 (12.77)	37.61 (10.17)^a^
	Control group, mean (SD)	43.83 (10.87)	43.08 (12.96)^a^
**Quality of life**			
	Intervention group, mean (SD)	0.66 (0.16)	0.76 (0.16)^a^
	Control group, mean (SD)	0.65 (0.24)	0.67 (0.25)^a^

^a^*P*<.001 (Intervention Group versus Control Group at week 4).

#### Performing Physiotherapy Exercises and Activities of Daily Self-Care

In both the intervention and control group, the patients’ ability to perform their physiotherapy exercises and daily self-care activities during the first four weeks after discharge increased. Patients in the intervention group performed better on each of the dimensions from the second week onwards (Table 6).

**Table 6:** Ability to perform physiotherapy exercises and activities of daily self-care.

	Performing physiotherapy exercises				Performing daily self-care activities			
	Intervention group, mean	Control group, mean	Model estimated difference (95% CI)	*P* value	Intervention group, mean	Control group, mean	Model estimated difference (95% CI)	*P* value
Week 1	6.54	6.52	0.01 (–0.53 to 0.51)	.98	6.73	6.30	–0.45 (–0.90 to 0.0)	.05
Week 2	7.28	6.53	–0.72 (–1.24 to –0.19)	.001	7.84	6.98	–0.83 (–1.28 to –0.37)	<.001
Week 3	7.35	6.93	–0.41 (–0.94 to 0.13)	.13	8.19	7.36	–0.79 (–1.26 to –0.33)	.001
Week 4	7.50	6.88	–0.60 (–1.12 to –0.08)	.03	8.32	7.64	–0.67 (–1.21 to –0.21)	.004

#### Satisfaction with Information and Patient-Perceived Involvement by the Hospital

In both the intervention and control group, patients’ satisfaction with the provided information and patients’ perceptions on how the hospital was involved in their recovery process decreased during the first four weeks after discharge. However, patients in the intervention group demonstrated a much smaller decrease over time (Table 7).

**Table 7:** Satisfaction with information and patient-perceived involvement by the hospital.

Week	Satisfaction with the Information				Patient-perceived involvement			
	Intervention group, mean	Control group, mean	Model estimated difference (95% CI)	*P* value	Intervention group, mean	Control group, mean	Model estimated difference (95% CI)	*P* value
1	7.90	7.40	–0.51 (–1.08 to –1.80)	.07	7.52	6.87	–0.74 (–1.36 to 0.11)	.002
2	7.96	6.61	–1.38 (–1.95 to –0.81)	<.001	7.75	6.14	–1.64 (–2.27 to –1.0)	<.001
3	7.45	6.16	–1.29 (–1.87 to –0.71)	<.001	7.23	6.13	–1.13 (–1.78 to –0.48)	<.001
4	7.61	5.32	–2.28 (–2.85 to –1.71)	<.001	7.24	4.90	–2.35 (–2.99 to 1.79)	<.001

#### Health Care Consumption

Patients in the intervention group had, on average, 1.22 points of contact with the hospital, their general practitioner (GP), or home-care organization compared to 1.62 points of contact in the control group, which is a 33% difference (*P*=.014). Of all contacts by the intervention group, 36% did not lead to an action (consultation or referral), compared to 45% in the control group, a 25% difference (*P*=.14) (Table 8).

**Table 8:** Health care consumption.

		Hospital	GP^a^	Home care	Total	Average points of contact^b^
**Intervention (n=105)**		69	34	25	128	1.22
	No action required	26	14	6	46	–^c^
**Control (n=90)**		59	58	28	145	1.62
	No action required	23	30	12	65	–

^a^GP: general practitioner.

^b^The average number of contacts with the hospital, GP, or home-care organization (corrected for the time points patients participated in the measurement).

^c^Not applicable.

### App Usage Data

In total, patients in the intervention group used the app 2418 times, which was an average of 26 times per patient. Most patients used a smartphone to access the information (75% vs 25% tablet use). The app was primarily used in the first 2 weeks, in which most of the information was offered (26/32 unique information items). Text-only information items related to pain, wound care, and self-care activities were consulted most frequently. Information about the start of the third and fourth week and reminders for anticoagulant usage and participation in study questionnaires were the least consulted information items.

Over the course of the four-week intervention, an average of 25 videos were offered to each patient. In total, these videos were viewed 2950 times, which was an average of 36 views per patient. Video-enriched information items related to physiotherapy exercises, pain, and wound care were viewed most frequently. Videos related to wearing a Thrombo-Embolic Deterrent (TED) hose, reminders for usage of anticoagulation medication, and information on what to expect in the fourth week were the least frequently viewed videos.

### Per Protocol Analysis

All results presented so far were analyzed using the intention-to-treat method. Analysis based on the per protocol method also resulted in the primary outcome (level of pain) being in favor of the intervention group, albeit somewhat more pronounced (Table 9).

**Table 9:** Per protocol analysis for level of pain.

Week	Pain at rest				Pain during activity				Pain during the night			
	Intervention group, mean	Control group, mean	Model estimated difference (95% CI)	*P* value	Intervention group, mean	Control group, mean	Model estimated difference (95% CI)	*P* value	Intervention group, mean	Control group, mean	Model estimated difference (95% CI)	*P* value
1	4.98	5.54	0.54 (–0.15 to 1.23)	.13	5.71	5.65	–0.20 (–0.64 to 0.68)	.95	5.95	6.11	0.16 (–0.55 to 0.87)	.65
2	4.37	5.38	1.08 (0.38 to 1.77)	.002	4.94	5.84	0.99 (0.33 to 1.66)	.003	5.20	6.01	0.90 (0.19 to 1.61)	.01
3	4.05	5.05	1.01 (0.30 to 2.80)	.001	4.65	5.16	0.63 (0.04 to 1.30)	.06	4.91	5.98	1.09 (0.38 to 1.81)	.002
4	3.31	4.59	1.26 (0.56 to 1.96)	<.001	3.83	5.08	1.34 (0.68 to 2.00)	<.001	4.04	5.21	1.18 (0.47 to 1.89)	.001

## Discussion

### Primary Findings

The results of our study demonstrate the effectiveness of using an app to actively educate patients on a day-to-day basis in the first four weeks of their recovery after TKR. Regarding the primary outcome, patients in the intervention group experienced a lower level of pain while at rest, during activity and at night. Furthermore, the app had a positive effect on physical functioning, quality of life, performing physiotherapy exercises and activities of daily self-care, satisfaction with the information, how the hospital was involved in the recovery process, and health care consumption. Finally, the intervention resulted in a trend towards less health care consumption.

To our knowledge, our study is the first to assess the effectiveness of an app to improve TKR patients’ self-management during the first four weeks of their recovery. In 2014, a Cochrane review on self-management programs for osteoarthritis showed that these programs (mainly including face-to-face and by-phone educational interventions) did not substantially improve self-management skills, pain, symptoms, physical functioning, or quality of life [29]. Furthermore, the authors concluded that new trials are unlikely to change the conclusions substantially, unless new models of self-management programs are introduced. In recent years, new self-management programs for patients (TKR included) have been introduced and have demonstrated the use and effectiveness of eHealth interventions, such as websites and portals [21]. An important difference between online interventions and app-based interventions like the one used in this trial is the ability to use push notifications. By using these notifications, information can be actively sent to patients at the time it is actually relevant to them instead of providing them with all the information all at once. Studies using apps in the TKR population have been performed but have focused on physiotherapy outcomes [28,30] and knowledge acquisition [31]. A 2018 multi-stakeholder analysis identified information needs during TKR treatment and suggested the use of technology to offer the right information at the right time [32]. As a result, patients would be allowed to better absorb the information and improve information recall and compliance. From a more clinical perspective, Filardo et al have suggested developing cointerventions to overcome kinesiophobia, the fear of physical activity due to pain. Kinesiophobia has significant impact upon patient recovery and final outcomes after TKR [33]. The app used in our study could be considered one of these cointerventions as it effectively lowered the level of pain in the intervention group.

With regards to economic benefits, using the app resulted in a decrease in health care consumption within the intervention group. This is in line with a 2017 review on the economic evidence for mobile health interventions, in which apps are reported to be an ideal platform for behavioral change in patients because of their popularity, connectivity, and increased sophistication when compared to text message or telephone follow-up [34]. A decrease in health care consumption is of clinical relevance, since the minimized length of stay for TKR has proven to increase the burden of care on hospital staff [35].

A major strength of our study was the holistic approach to the early-phase recovery of TKR patients, combining insights from orthopedic surgeons, physiotherapists, and (specialized) nurses. In addition, historical data on TKR health care consumption was used to form an interactive timeline to guide patients during their recovery. Using push notifications to actively alert patients about newly available information resulted in an average use of the app of 26 times per patient. Additionally, the usage of short, one subject only videos has proven to be of added value, as patients watched many of the 25 available videos multiple times. This was especially the case with videos related to pain, wound care, and physiotherapy exercises, demonstrating patients’ needs for this type of information.

A limitation of our study is the number of patients in the intervention group that did download and use the app. Out of the 114 possible app users, 93 patients downloaded the app (82%). This demonstrates the necessity of assessing a patient’s digital health literacy and supporting them during the initial usage of interventions like these. Nevertheless, a per protocol analysis showed similar results. Another limitation could be the usage of self-developed questions which might introduce a risk of bias. To minimize this, questions were developed together with health care providers, were screened for readability by a specialized organization and were evaluated by several patients. Finally, we did not consider direct patient feedback when developing the content for the app but instead based it solely on the knowledge and experience of hospital staffs. In future research, patients should have a more prominent role to play in the development and evaluation of the app’s (patient-specific) content, the desired timing of notifications, and the preferred mode of information to further optimize outcomes. In addition, future research could focus on the generalizability of interventions like these in other treatments, as well as their cost-effectiveness.

### Conclusion

In conclusion, we found that, in comparison with standard patient education, the active education and coaching of patients on a day-to-day basis via the app in the four weeks after TKR resulted in a significant decrease in patients’ levels of pain. Additionally, there was a significant improvement in patients’ physical functioning, quality of life, their ability to perform physiotherapy exercises and activities of daily self-care, their satisfaction with the information, their perceived involvement by the hospital, and their health care consumption. Given the rising number of TKR patients and the increased emphasis on self-management, we suggest using an app with timely postoperative care education as a standard part of care.

## Appendix

Multimedia Appendix 1 Overview of information offered to patients in the intervention group.

Multimedia Appendix 2 Self-developed questions for the study.

Multimedia Appendix 3 Consort eHealth checklist.

## Abbreviations

ANOVA: analysis of variance
CONSORT: Consolidated Standards of Reporting Trials
eHealth: electronic health
GP: general practitioner
mHealth: mobile health
NRS: Numeric Rating Scale
TED: Thrombo-Embolic Deterrent
TKR: total knee replacement
