# Supplementary material for: The Effect of an App for Day-to-Day Postoperative Care Education on Patients With Total Knee Replacement: Randomized Controlled Trial
Source: JMIR Mhealth Uhealth. 2019 Oct 21;7(10):e15323. doi: 10.2196/15323 (PMC6914303; doi:10.2196/15323)
Supplement: Multimedia Appendix 1 [file mhealth_v7i10e15323_app1.pdf]

Appendix I: Overview of information offered to patients in the intervention group *

#	Days after discharge **	Push-notification	Topic ***	Mode of information	Video presenter
1	0	Yes, 9 am	Take a photo of your medication protocol to make sure you have it available on your smartphone or tablet at all times.	Text	
2	1	Yes, 1 pm	Welcome back at home. With this app we will educate and coach you in the first 4 weeks after your surgery.	Text, video	Orthopedic surgeon
3	1	Yes, 3 pm	In the first weeks after surgery patients experience a quite some of pain. This is normal, given the fact that you have underwent major surgery. You don't need to worry about this.	Text, video	Orthopedic surgeon
4	1	Yes, 5 pm	From the first day onwards, exercises are an important part of your recovery, even though you have just had surgery and your knee is still painful and swollen.	Text, video	Physiotherapist
5	1	No	These videos show you an example of all the exercises: how to perform them and the number of repetitions.	Text, video	Physiotherapist
6	1	No	During your recovery you might need to use crutches. This video demonstrates walking with crutches and getting in and out of a chair while using crutches.	Text, video	Physiotherapist

7	1	Yes, 8 pm	This was the first day of your recovery. Hopefully you have been able to manage your pain a little. Be sure to take your pain medication before going to sleep.	Text, video	Orthopedic surgeon
8	2 **** (5)	Yes, 11 am	As soon as you are at home, you are allowed to take a shower. Don't use shampoo near the wound. Be sure to dry pat the wound.	Text, video	Physician assistant / nurse
9	2 **** (6,9,13, 16, 19, 23, 26)	Yes, 2 pm	Please provide us with a score, reflecting your amount of pain at this moment.	Text, Numeric scale (0-10)	
10	2	Yes, 8 pm	Patients often worry about their level of pain. Please remember you have only recently had surgery. This kind of surgery comes with pain and limitations in movement in the first week weeks.	Text, video	Orthopedic surgeon
11	3	Yes, 10 am	Make sure you perform the exercises several times a day. You might experience pain while doing so, this is normal.	Text, video	Physiotherapist
12	3 **** (10)	Yes, 1 pm	Please make sure to contact the hospital directly when you have a fever or when the wound becomes more painful or starts to leak	Text, button to call the hospital	
13	3 **** (6)	No	Today's videos show how the exercises are performed.	Text, video	Physiotherapist

14	3	Yes, 4 pm	During the course of this first week, we will contact you by phone. Please make sure to write down your questions or concerns.	Text, video	Physician assistant / nurse
15	4 **** (11,18)	Yes, 11 am	Already in the hospital you have started to use blood thinning medication (anticoagulantia). It is important to keep taking the medication in the 4 weeks after surgery.	Text, video	Orthopedic surgeon
16	4	Yes, 4 pm	After surgery, stitches have been used to close the wound. After about 2 to 3 weeks after discharge, these will be removed at the hospital.<br><br>OR<br><br>After surgery, glue and tapes strips have been used to close the wound. After about l0 days you can remove these yourself.	Text, video	Physician assistant / nurse
17	4	Yes, 4 pm	As a result of the surgery, you might see hemorrhages on your upper or lower leg. You don’t need to worry about this.	Text, video	Physician assistant / nurse
18	5	Yes, 11 am	Patients often worry about the healing of the knee. In this phase of your recovery it is normal to experience pain and swelling of your knee.	Text, video	Orthopedic surgeon
19	5	Yes, 5 pm	While performing the exercises, you might experience some pain. This is normal. Make sure the	Text, video	Physiotherapist

			pain decreases in the hours after performing the exercises.		
20	6	Yes, 11 am	If you are thinking about phasing out your pain medication, be sure to start with the strongest, as they often have most side effects. When, as a result, the pain becomes too severe, you can still take the pain killer you stopped with.	Text, video	Orthopedic surgeon
21	6 **** (13,20,27)	Yes, 5 pm	Tomorrow you will receive the questionnaires for the study. Of course, we hope you are willing to participate in these.	Text	
22	7	Yes, 5 pm	You have been at home for about a week now. Do you remember your first days? We hope you experience less pain and are better capable of performing the exercises.	Text, video	Orthopedic surgeon
23	7 **** (14,21,28)	Yes, 8 pm	During the course of your recovery, we ask you to provide us with your pain scores. This graph shows the progression of your level of pain so far.	Text, graph	
24	8	Yes, 11 am	Today is the start of the second week of coaching you with our app. Don’t worry too much if you still experience pain, this is uncomfortable but normal.	Text, video	Orthopedic surgeon
25	9 **** (16)	Yes, 11 pm	If you have an indication for using compression stockings, also referred to as a TED hose, make sure to wear it until 4 weeks after surgery.	Text, video	Orthopedic surgeon

26	10	Yes, 11 pm	Do you still have enough pain medication? If not, please contact the hospital to get a refill prescription.	Text	
27	11 **** (16)	Yes, 3 pm	Please check the wound on your knee. It needs to be dry at this moment. If it is leaking, please make sure to contact the hospital.<br><br>OR<br><br>Please check the wound on your knee. It needs to be dry at this moment. If it is leaking, please make sure to take a photo with this app. The app will send the image to us right away.	Text, video	Orthopedic surgeon
28	14	Yes, 11 am	Before you decide to participate in traffic again, please make sure to discuss this with your physio therapist.	Text, video	Physiotherapist
29	15	Yes, 11 am	Today is the start of the third week of coaching you with our app. Don't worry too much if you still experience pain, this is uncomfortable but normal.	Text, video	Orthopedic surgeon
30	22	Yes, 11 am	Today is the start of the fourth week of coaching you with our app. This is also the last week in which we do so.	Text, video	Orthopedic surgeon
31	28	Yes, 5 pm	Today was the last day of support though this app. We hope you enjoyed using the app. Please continue performing your exercise in the coming weeks.	Text, video	Orthopedic surgeon

			We will see each other soon at our hospital for your check-up consultation.		

* Provisioning of information differed slightly between participating hospitals; this table provides a generic overview.

** 0 days after discharge refers to the day of discharge itself

*** This column provides a general description of the information offered.

**** This information is offered more than once. The days on which it is repeated is indicated between parentheses.
