# Supplementary material for: The Effect of an App for Day-to-Day Postoperative Care Education on Patients With Total Knee Replacement: Randomized Controlled Trial
Source: JMIR Mhealth Uhealth. 2019 Oct 21;7(10):e15323. doi: 10.2196/15323 (PMC6914303; doi:10.2196/15323)
Supplement: Multimedia Appendix 2 [file mhealth_v7i10e15323_app2.pdf]

Appendix II: Self-developed questions for the study

PAIN

1. What amount of knee pain (operated side) have you experienced in **the last** week while **at rest?**

No pain at all 0 1 2 3 4 5 6 7 8 9 10 Worst pain imaginable

2. What amount of knee pain (operated side) have you experienced in **the last** week **during activity?**

No pain at all 0 1 2 3 4 5 6 7 8 9 10 Worst pain imaginable

3. What amount of knee pain (operated side) have you experienced in **the last** week **during the night?**

No pain at all 0 1 2 3 4 5 6 7 8 9 10 Worst pain imaginable

SATISFACTION WITH INFORMATION

1. To what extend were you satisfied with the information you received from the hospital **last week**, with

regards to your recovery**?**

Not satisfied at all 0 1 2 3 4 5 6 7 8 9 10 Very much satisfied

SATISFACTION WITH HOSPITAL INVOLVEMENT IN RECOVERY

1. To what extend were you satisfied with the level of involvement by the hospital **last week**, with

regards to your recovery**?**

Not satisfied at all 0 1 2 3 4 5 6 7 8 9 10 Very much satisfied

SELF-CARE

1. To what extend were you able to take care of yourself **last week**?

Not at all 0 1 2 3 4 5 6 7 8 9 10 Very much

HEALTH-CARE CONSUMPTION

1. Did you contact the hospital last week, because of your knee (operated side)? Yes / No

If Yes: What was the outcome of the contact?

a. No action or follow-up required

b. Follow-up required (new medication, consultation booked, direct admission to the hospital)

2. Did you contact your GP last week, because of your knee (operated side)? Yes / No

If Yes: What was the outcome of the contact?

a. No action or follow-up required

b. Follow-up required (new medication, consultation booked, referral to the hospital)

3. Did you contact the home care organization last week, because of your knee (operated side)? Yes / No

If Yes: What was the outcome of the contact?

a. No action or follow-up required

b. Follow-up required (consultation booked, referral to the GP)
